# Recent advances in engineering the central carbon metabolism of industrially important bacteria

**DOI:** 10.1186/1475-2859-11-50

**Published:** 2012-04-30

**Authors:** Maria Papagianni

**Affiliations:** 1 Department of Hygiene and Technology of Food of Animal Origin, School of Veterinary Medicine, Aristotle University of Thessaloniki, Thessaloniki, 54006, Greece

## Abstract

This paper gives an overview of the recent advances in engineering the central carbon metabolism of the industrially important bacteria *Escherichia coli*, *Bacillus subtilis*, *Corynobacterium glutamicum*, *Streptomyces* spp., *Lactococcus lactis* and other lactic acid bacteria. All of them are established producers of important classes of products, e.g. proteins, amino acids, organic acids, antibiotics, high-value metabolites for the food industry and also, promising producers of a large number of industrially or therapeutically important chemicals. Optimization of existing or introduction of new cellular processes in these microorganisms is often achieved through manipulation of targets that reside at major points of central metabolic pathways, such as glycolysis, gluconeogenesis, the pentose phosphate pathway and the tricarboxylic acid cycle with the glyoxylate shunt. Based on the huge progress made in recent years in biochemical, genetic and regulatory studies, new fascinating engineering approaches aim at ensuring an optimal carbon and energy flow within central metabolism in order to achieve optimized metabolite production.

## Introduction

Metabolic engineering is a field that encompasses detailed metabolic analysis aiming at identifying targets for manipulation through recombinant DNA technology for the improvement and/or design of cells [1]. Metabolic engineering is principally concerned with understanding the regulation of metabolic systems as a whole.

The term “central carbon metabolism” (CCM) describes the integration of pathways of transport and oxidation of main carbon sources inside the cell. In most bacteria, the main pathways of the CCM are those of the phosphotransferase system (PTS), glycolysis, gluconeogenesis, pentose phosphate (PP) pathway, and the tricarboxylic acid cycle (TCA) with the glyoxylate bypass. As a whole, the system has a complex structure and it is regulated by complex networks of reactions. The knowledge about regulation in CCM has great industrial relevance as it may allow the engineering of selected metabolic steps to reroute carbon fluxes toward precursors for industrially important metabolites [2]. This kind of metabolic engineering however is a difficult task as there is still significant lack of knowledge regarding the regulation of central carbon metabolism flux for many industrially important bacteria. *Escherichia coli* is by far the most extensively studied bacterium in this field. Most recently acquired knowledge provides fundamental insights into the regulation of fluxes in CCM through transcriptional control [3], a finding that may hold for other bacteria as well, and is expected to have a significant impact in industrial biotechnology.

The field of metabolic engineering is broad and diverse and rapidly expanding and the reader will find in the literature a number of excellent reviews that cover the area from different perspectives. The present, aims to give an overview of the recent advances in engineering the CCM of the industrially important bacteria *E. coli*, *Bacillus subtilis*, *Corynobacterium glutamicum*, *Streptomyces* spp., *Lactococcus lactis* and other lactic acid bacteria. All of them are established producers of important classes of products, e.g. proteins, amino acids, organic acids, antibiotics, high-value metabolites for the food industry and also, promising producers of a large number of industrially or therapeutically important chemicals. Optimization or introduction of new cellular processes in these microorganisms is often achieved through manipulation of targets that reside at major points of central metabolic pathways. Only approaches that involve manipulations of such points will be discussed in this review.

### Escherichia coli

*E. coli*, a Gram-negative bacterium, is being used widely today in a large number of biotechnological processes. The ease of cultivation –it grows quickly in minimal media, as well as its ability to metabolize both pentoses and hexoses [4], have made it the bacterium of choice for research and over the years the wealth of information in genomics, proteomics and metabolism have led it to be regarded as the prime prokaryotic model [5]. Many feasible genetic tools have been developed and the cloning methodologies for *E. coli* developed for the production of foreign proteins established it as a “cell-factory”. *E. coli* is the organism of choice for the expression of a wide variety of recombinant proteins for industrial, therapeutic and diagnostic applications. Apart from proteins, *E.coli* has been shown to be a suitable host for the production of many other valuable metabolites.

**Figure 1:**
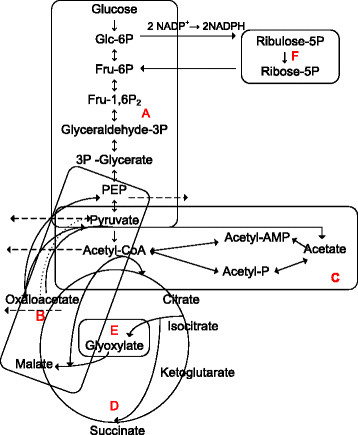
**Simplified representation of the central carbon metabolism of*E. coli*. **(A) glycolysis and gluconeogenesis, (B) anaplerotic reactions, (C) acetate formation and assimilation, (D) TCA cycle, (E) Glyoxylate shunt, (F) PP pathway. The dotted line arrow from oxaloacetate to pyruvate indicates the anaplerotic reaction catalysed by pyruvate carboxylase. The broken line arrows indicate the removal of metabolites.

The CCM of *E. coli* and specifically the metabolism of glucose are intensively studied and well known topics [6,7]. Glucose metabolism starts with its uptake via the PTS and proceeds with several interconnected pathways with the major being: glycolysis, gluconeogenesis, the pentose-monophosphate bypass with the Entner-Dudoroff pathway, the TCA cycle with the glyoxylate bypass, anaplerotic reactions and acetate production and assimilation (Figure 1). The network controlling the carbon uptake integrates metabolism, signal transduction and gene expression [8]. The extensive knowledge gained on *E. coli* CCM [6] offers key advantages in metabolic engineering efforts that aim at achieving increased metabolite production. Such efforts have been focused intensively rather on the upper part of the carbon assimilation network, consisting of glycolysis and glyconeogenesis and their genetic and metabolic regulation.

The terminal stages of glycolysis in *E. coli* involve complex interplays. PEP conversion is coupled to two metabolic processes: PEP may give rise to pyruvate by pyruvate kinase (PK) in the PTS or it may give rise to oxaloacetate by the PEP carboxylase (*ppc*)-catalyzed anaplerotic reaction.

Pyruvate, the end-product of glycolysis, is oxidized to acetyl-CoA and CO_2_ by the pyruvate dehydrogenase complex. Acetyl-CoA participates in a large number of reactions either as substrate or as product: it can enter the TCA cycle, it can be used in fatty acid and triglycerides biosynthesis and it can be used in acetate biosynthesis. Acetyl-CoA connects the glycolysis and the acetate metabolism pathways with the TCA cycle and the glyoxylate shunt. Acetyl-CoA is therefore a key factor in determining biomass formation, the redox balance and energy yield. Moreover, the acetate-acetyl-CoA node determines a large part of the control exerted by central metabolism on the performance of many microbial processes [9].

The final products of glycolysis PEP and pyruvate enter the TCA cycle via acetyl-CoA and via the formation of oxaloacetate by carboxylation. This route, referred to as anaplerosis, replenish the intermediates of the TCA cycle that were used for anabolic purposes. Under gluconeogenic conditions, the TCA cycle intermediates oxaloacetate or malate are converted to pyruvate and PEP by decarboxylation and this way the PEP-pyruvate-axaloacetate node provides the precursors for gluconeogenesis. Therefore, the metabolic link between glycolysis, gluconeogenesis and the TCA cycle is represented by the PEP-pyruvate-oxaloacetate node [6].

Key points of the above processes that represent main problems related to the use of *E. coli* as a cell factory are the following: a) PEP availability as a building block for the production of other valuable compounds is reduced; b) acetyl-CoA accumulation can affect the utilization of glucose by causing accumulation of pyruvate and enhancing acetate production; and c) acetate accumulation could lead to a serious waste of carbon as up to 1/3 of the glucose used could appear as acetate.

Acetate co-production by *E. coli* in recombinant protein fermentations represents a serious problem that has been discussed by many authors [10]. Apart from the diversion of carbon that might otherwise be used in synthesis of biomass or the protein product, acetate production causes a number of other problems since it inhibits growth even at very low concentrations, e.g. 0.5 g/l [11], it inhibits protein formation [12], it affects proteins and genes involved in stress response and regulation processes [13], it interferes with methionine biosynthesis [14], it interferes with bacterial energetics as it dissipates the pH component of the membrane potential, and it causes pH control problems in fermentation [15]. It has been shown that acetate is more inhibitory to recombinant protein producing strains than to wild-type ones as the threshold growth rate for the onset of acetate production is lower in the first [16]. Thus, control of acetate formation is of paramount importance in recombinant protein production systems with *E. coli*.

The rate at which acetate forms is directly related to the growth rate and the glucose consumption rate. Since in the common for *E. coli* operational mode of fed-batch culture, the growth rate is determined by the feeding rate of the limiting substrate, *E. coli* generates acetate when glucose is the limiting substrate and cells grow above a threshold growth rate [10]. In the applied fully aerobic conditions, the availability of oxygen is not related to the formation of acetate however, the rate of oxygen uptake is the factor that determines it and most process and genetic engineering strategies aim to facilitate the appropriate balance between growth rate and oxygen uptake.

Acetate formation is influenced by operational aspects of *E. coli* processes e.g. feeding strategies in fed-batch fermentations, and by the composition of the culture medium. There have been many attempts to control acetate formation by process modifications that mainly aim to control the growth rate of the microorganism [10]. These approaches will not be discussed in the present review. Similarly, there have been many attempts to reduce acetate formation by genetic engineering that use different approaches and strategies. These strategies focus either on the uptake and consumption rates of glucose or on the flux from the central pathways to acetate or finally on the metabolic and regulatory mechanisms that lead to accumulation of acetate.

Picon et al. [17] studied the effects of changes made in the glucose uptake capacity on the flux distribution to the desired end product β-galactosidase and to acetate. The lack of one of the components (IICB(Glc) protein) of the glucose-PTS reduced the growth rate significantly. However, β -galactosidase production had no effect on growth rate. The constructed strain directed more carbon into biomass and carbon dioxide, and less into acetate. β -galactosidase was produced in amounts not significantly different from the wild-type strain from half the amount of glucose.

De Anda et al. [18] studied the effect of replacing the glucose-PTS with an alternate glucose transport activity on growth kinetics, acetate accumulation and production of two model recombinant proteins. The engineered strain maintained similar production and growth rate capabilities while reducing acetate accumulation. Specific glucose consumption rate was lower and product yield on glucose was higher in the engineered strain in fed-batch culture. Altogether, strains with the engineered glucose uptake system showed improved process performance parameters for recombinant protein production over the wild-type strain.

The approach of Wong et al. [19] to reducing acetate accumulation was to disable the PEP:PTS (phosphoenelopyruvate:phosphotransferase system) by deleting the ptsHI operon in the wild-type *E. coli strain* GJT001. The mutation caused a severe reduction in growth rate and glucose uptake rate in glucose-supplemented minimal medium, which confirmed the mutation, and eliminated acetate accumulation. The mutant strain apparently metabolized glucose by a non-PTS mechanism followed by phosphorylation by glucokinase. The ptsHI mutant of GJT001 resulted in reduced acetate accumulation, which led to significant improvements in recombinant protein production in batch bioreactors.

Lara et al. [20] worked with an *E. coli* strain lacking the PEP:PTS, at glucose concentrations of up to 100 g/L in batch mode and showed that high cell and recombinant protein concentrations are attainable in simple batch cultures by circumventing overflow metabolism through metabolic engineering. At the highest glucose concentration tested, acetate accumulated to a maximum of 13.6 g/L for the parental strain whereas a maximum concentration of only 2 g/L was observed for the engineered strain. This represents a novel and valuable alternative to classical bioprocessing approaches.

The main route for acetate production is from acetyl-CoA through acetyl-phosphate by the two enzymes phosphotransacetylase (*pta*) and acetate kinase (*ack*). The second approach of redirecting the flow of carbon to acetate may involve the elimination of the activity of these two enzymes as described in the works of Bauer et al. [21], Hahm et al. [22], and Chang et al. [23]. In a similar approach, Kim and Cha [24] reported the use of an antisense RNA strategy to partially block the synthesis of *pta* and *ack* and the resulted improved recombinant protein production. However, in the recent work of Castano-Cerezo et al. [9], deletion of *pta* was shown to strongly affect the expression of several genes related to central metabolic pathways, as well as the ability of *E. coli* to grow on acetate under anaerobic conditions due to decreased acetyl-CoA synthetase, glyoxylate shunt and gluconeogenic activities.

As the accumulation of pyruvate signals the onset of acetate formation, in the third approach that focuses on the metabolic and regulatory mechanisms that lead to accumulation of acetate, either pyruvate or the glycolytic products should be diverted to where carbon is needed: in the TCA cycle. Vemuri and co-workers [25] showed that several genes involved in the TCA cycle and respiration are repressed as the glucose uptake rate increases and deletion of the gene coding for the regulatory protein ArcA resulted in reduction of the produced acetate and increase of biomass due to the increased capacities of the TCA cycle and the respiratory chain.

The diversion of carbon from acetate or its precursors to the TCA cycle can be accomplished by manipulating the expression levels of the anaplerotic enzyme PEP carboxylase. Overexpressing the native PEP carboxylase reduced acetate formation by 60 % in shake flasks cultures grown at low cell density and significantly increased biomass yield [26,27]. However, PEP carboxylase (*ppc*) overexpression diminished the supply of PEP needed for the PTS-mediated uptake of glucose and decreased the growth rate of the organism. The anaplerotic function of PEP carboxylase was also shown to be fully restored by expression of pyruvate carboxylases in *ppc* mutants [28]. The result was an increased carbon flux to oxaloacetate during growth of *E. coli* on glucose under both aerobic and anaerobic conditions [28,29]. Under aerobic conditions, the flux redirection resulted in a 50 % increase in yield of biomass while glucose uptake and specific growth rates remained unaltered. Similarly, March and co-workers [30] in their work with an *E. coli* strain containing pyruvate carboxylase (*pyc*) reported a 60 % decrease of acetate while the protein yield increased by 68 %, while Vemuri et al. [31] reported an 80 % reduction of acetate upon introduction of heterologous pyruvate carboxylase.

Acetate formation is affected by the NADH/NAD ratio. Vemuri et al. [32] reported the complete elimination of acetate and a 10 % increase in protein yield in an *arcA* mutant by reducing the redox ratio through expression of the water-forming NADH oxidase from *Streptococcus pneumoniae*.

The PEP-pyruvate-oxaloacetate node [6] is receiving increased attention and this is mostly driven by the need to manipulate the carbon flux through the node for improved production in various biotechnological processes. Such a case is the production of succinate by *E. coli* that involves extensive anaplerotic fluxes. Various metabolic engineering approaches have been implemented successfully, including everexpression of the native *ppc*[33], the NAD-dependent malic enzyme ScfA [34], a heterologous PEP carboxykinase (*pck*) [35], use of alternative glucose transport systems to increase energy efficiency by eliminating the need to produce additional PEP from pyruvate, a reaction that requires two ATP equivalents [36] and combined approaches such as overexpression of heterologous *pck* and inactivation of the PEP-dependent PTS [37]. Another strategy for the efficient conversion of glucose to pyruvate was to combine mutations to minimize ATP yield, cell growth and CO_2_ production with mutations to eliminate acetate production [38].

An important focus area for metabolic engineering of the PEP-pyruvate-oxaloacetate node is the production of aromatic compounds. The shikimic acid (SA) pathway is the common route leading to the biosysnthesis of aromatic compounds in bacteria as well as in several eukaryotic organisms and plants [39]. In *E. coli*, the first step in this pathway is the fusion of PEP precursors and erythrose-4P into 3-deoxy-D-arabinoheptulosonate-7P (DAHP) by the DAHP synthase [40]. Since PEP is also required for the PTS-glucose uptake and for anaplerosis, it is obvious that enhancing PEP availability is necessary for high-level production of aromatic compounds in *E. coli*. Metabolic engineering approaches that aimed successfully at increased PEP availability include inactivation of the PTS operon, installation of non-PTS glucose transporters like glucose facilitators, and transformation with plasmids that carry the *tktA* and *ppA* genes, coding for transketolase I and PEP synthase, respectively, in order to increase availability of the intermediates E4P and PEP [41-44]. Combining CCM modifications with SA pathway modifications, like partial or total blockage of the SA flux into chorismic acid (CHA), e.g. by inactivating *aroK* and *aroL* genes, resulted in significant increases in SA [45] or anthranilate accumulation [46].

The metabolic network of the TCA cycle and the glyoxylate bypass in *E. coli* has been the focus of various engineering strategies during the last decade due to the tremendous increase of interest for the efficient production of high-value platform chemicals and building blocks for bio-based polymers. Succinic acid is a platform chemical that can be converted to 1,4-butanediol, γ-butyrolactone, tetrahydrofuran, and other chemicals of industrial importance. Succinic acid formation occurs primarily through the reductive branch of the TCA cycle (fermentative pathway) during anaerobic conditions, and the glyoxylate bypass. However, due to insufficient reducing power, wild-type *E. coli* can produce only a small amount of acid (7.8 %) through the fermentative pathway [47,48]. Succinic acid production was improved either by introducing genetically modified metabolic pathways that enhance key enzyme activity or by deleting or inhibiting competitive to succinic acid pathways [49]. Successful examples of the first strategy include the overexpression of *ppc*[33], of heterologous *pck*[35], of *pyc*[50], that led to significant increases -even 6.5-fold-in the production of succinate. A successful example of the second strategy involves the deactivation of *αdhE* (aldehyde dehydrogenase), *ldhA* (lactate dehydrogenase) and *αck-ptα* (acetate kinase-phosphate acetyltransferase) from the central metabolic pathway and activation of the glyoxylate bypass through the inactivation of *iclR* that encodes a transcriptional repressor protein of the glyoxylate bypass [51]. Lin et al. [47] constructed a mutant *E. coli* strain by deletion of *αck-ptα, iclR*, and *ptsG* genes and overexpression of the *ppc* gene and achieved high-level succinate production under aerobic conditions.

The CCM includes also the pentose-phosphate (PP) pathway. In the oxidative branch of the pathway which supplies reducing power for cellular biosynthetic processes, NADPH is generated via oxidation of glucose-6-phosphate (G-6P) by G-6P dehydrogenase (G6PDH, encoded by *zwf*). The non-oxidative branch includes the interconversion of ribulose-5P, ribose-5P and xylulose-5P and the transfer of either a glycoaldehyde group or a dihydroxyacetone group among sugar phosphates by transketolase and transaldolase, respectively. Engineering the PP pathway has received attention either for the redirection of the carbon flux to mainstream glycolytic pathway or for overproduction of NADPH. Knocking out *zwf* resulted in a non-operational PP pathway and an increased TCA activity in the work of Zhao et al. [52]. Few studies however have been focused on G-6P dehydrogenase as a key enzyme that activates the PP pathway. Homologous co-overexpression of *zwf* and *glpX* (encodes for FBPase II) activated the PP pathway and increased the NADPH-dependent hydrogen production to 2.32-fold [53]. Overproduction of NADPH is of primal importance for various synthetic pathways e.g. for the efficient production of poly(3-hydroxybutyrate) (PHB). Since NADPH is mainly formed in the PP in *E. coli*, knocking out phosphoglucose isomerase (*pgi*) forced the carbon flow into the PP pathway and enhanced NADPH production but repressed cell growth [54]. Cell growth was recovered to some extend by introducing a NADPH-consuming pathway, such as the PHB synthetic pathway and efficient PHB production was achieved by appropriately controlling the glucose concentration of the substrate.

### Bacillus subtilis

*Bacillus* spp. are used industrially for the production of enzymes (amylases, lipases, proteases), vitamins, antibiotics, purine nucleotides, poly-γ-glutamic acid, d-ribose, PHB and other metabolites. *B. subtilis* is able to metabolize a wide range of carbohydrates, including monosaccharides (e.g., glucose, xylose), oligosaccharides (e.g., maltodextrins, cellodextrins), and polysaccharides (e.g., starch)- but not cellulose [55,56]. *B. subtilis* has been an attractive host for the expression of foreign proteins (e.g. enzymes) on an industrial scale due to its ability to transport them out of the cell through secretion systems.

The TCA cycle is one of the major routes of carbon catabolism in *B. subtilis*[57]. Anaplerotic reactions contribute to the flux through the TCA cycle. The reactions at the interface between the lower part of glycolysis and the TCA cycle were identified as an important metabolic subsystem in this organism [58].

*B. subtilis* is an important model microorganism in the field of metabolic engineering for the production of riboflavin [59]. The PP pathway and the pyruvate shunt were identified as major pathways of glucose catabolism in a recombinant riboflavin-producing *B. subtilis* strain [60]. Reactions connecting the TCA cycle and glycolysis, catalyzed by the malic enzyme and PEP carboxykinase were found to consume up to 23 % of the metabolized glucose. The overall flux distribution suggested that *B. subtilis* metabolism has an unusually high capacity for the reoxidation of NADPH. Under the conditions investigated [60], riboflavin formation in *B. subtilis* is limited by the fluxes through the biosynthetic rather than the central carbon pathways, suggesting a focus for metabolic engineering of this system. Therefore, overexpression of enzymes (e.g. G6PDH and 6PGDH) that facilitate the route of carbon flow towards the PP pathway increased the pool of ribulose-5P which is a precursor for riboflavin biosynthesis and led to increased riboflavin yields [61-63].

It is expected that *B. subtilis* will play an important role in the process of converting biomass into biocommodities [64,65]. In this view, modifications of the heterofermentative and homofermentative metabolism of the organism have been reported recently, along with the construction of recombinant cellulolytic strains. Romero and co-workers [66] identified a key physiological role in LDH for glucose consumption under heterofermentative metabolism. By inactivating the *ldh* gene and eliminating butanediol biosynthesis, the resulting recombinant *B. subtilis* strain produced enthanol as the sole fermentation product. To increase L-lactate production, Romero-Garcia and co-workers [67] constructed the *B. subtilis* CH1 *alsS*^-^ strain that lacks the ability to synthesize 2,3-butanediol. Inactivation of the pathway that competed for pyruvate availability, led to a 15 % increase in L-lactate yield from glucose compared with the parental strain. Zhang et al. [68] proceeded in the direct production of lactate from cellulose as the sole carbon source without any other organic nutrient by recombinant cellulolytic *B. subtilis.* Overexpression of the endoglucanase BsCel5 enabled *B. subtilis* to grow on solid cellulosic materials as the sole carbon source for the first time. Furthermore, the specific activity of BsCel5 on regenerated amorphous cellulose (RAC) and its expression/secretion level in *B. subtilis* was increased by two-round directed evolution enhance. To increase lactate yield, the alpha-acetolactate synthase gene (*alsS*) in the 2,3-butanediol pathway was knocked out.

### Corynobacterium glutamicum

The Gram-positive bacterium *C. glutamicum* was discovered 50 years ago as a natural overproducer of glutamate. Today, it is used for the industrial production of more than 2 million tons of amino acids (glutamate, lysine and tryptophan) per year [69]. Decades of intensive research triggered by the economical importance of the microorganism, highlighted its metabolism [70-73] and provided the complete genome sequence [74] along with efficient genomic approaches and metabolic engineering strategies aiming at novel and advanced bioprocesses. The recent review by Wittmann [69] provides a rich source of information on metabolic engineering strategies applied to *C. glutamicum* over the last years.

The CCM in *C. glutamicum* involving glycolysis, the PP pathway, the TCA cycle and the anaplerotic and gluconeogenic reactions are intensively studied and well known topics today [69]. The PP pathway and its role in NADPH regeneration for lysine biosynthesis has been the subject of a number of important studies. The PP pathway in *C. glutamicum* is mainly regulated by the ratio of NADPH/NADP concentrations and the activity of G6PDH [75]. Together with G6PDH, 6PGDH and isocitrate dehydrogenase, they represent the main NADPH sources in this organism [76]. Intracellular metabolite concentrations and specific enzyme activities during lysine overproduction in two isogenic leucine auxotrophic strains, which differed only in the regulation of their aspartate kinases (a key enzyme of the lysine production pathway), were compared by Moritz et al. [76]. Analyses confirmed that NADPH regeneration in the PP pathway is essential for lysine biosynthesis in *C. glutamicum*. Marx et al. [77] constructed a phosphoglucose isomerase (*pgi*) mutant strain of *C. glutamicum* to redirect the carbon flux through the PP pathway. L-lysine production increased as well as by-product formation and growth rate, a common feature of *pgi* mutants due to disturbed metabolism of NADPH.

Various approaches have aimed at increasing the flux through the PP pathway. Overexpression of the *fbp* gene, encoding fructose 1,6-bisphosphatase, increased lysine yield on glucose, sucrose and fructose up to about 40 % by enhancing the PP pathway flux by 10 % [78]. Another major target approached was the *zwf* gene, encoding G6PDH, the overexpression of which resulted in increased lysine production on different carbon sources [79]. Modification of the regulatory properties of the PP pathway enzymes led also to significant increases of the flux through the PP pathway [79]. Introduction of 12 defined genome-based changes in genes encoding central metabolic enzymes redirected major carbon fluxes as desired towards the optimal pathway usage predicted by in silico modelling in the work of Becker et al. [80]. The engineered *C. glutamicum* produced lysine with a high yield of 0.55 g /g glucose, a titer of 120 g /L lysine and a productivity of 4.0 g /L/h in fed-batch culture. The specific glucose uptake rate of the wild type was completely maintained during the engineering process, providing a highly viable producer. This is the first report of a rationally derived lysine production strain that may be competitive with industrial applications.

For anaplerotic replenishment of the TCA cycle, *C. glutamicum* exhibits-in contrast to many other organisms- pyruvate carboxylase (*pyc*) and PEP carboxylase (*ppc*) as carboxylating enzymes [73]. In addition to the activities of the above C3-carboxylating enzymes, *C. glutamicum* possesses three C4-decarboxylating enzymes converting oxaloacetate or malate to PEP or pyruvate. The individual flux rates in this complex anaplerotic node were investigated by using ^13^ C]-labelled substrates. The results revealed that both carboxylation and decarboxylation occur simultaneously in *C. glutamicum* and a high cyclic flux of oxaloacete via PEP to pyruvate was found [73]. The PEP-pyruvate-oxaloacetate node in *C. glutamicum* has been discussed extensively in the review by Sauer and Eikmanns [6]. Since a variety of amino acids originate their biosynthesis from the PEP-pyruvate-oxaloacetate node, the enzymes in this node and in particular the anaplerotic enzymes have been regarded as important targets for metabolic engineering of *C. glutamicum.* Overexpression of *pyc* resulted in seven-fold increase of glutamate production as well as increased lysine accumulation, while abolition of pyruvate carboxylase activity resulted in significantly lower amino acid formation [72]. “Genome-based strain reconstruction” confirmed the role of *pyc* in lysine production [81]. However, Koffas et al. [82] showed that overexpression of *pyc* increased growth but decreased the specific productivity of lysine. Simultaneous expression of aspartate kinase abolished the deficiency and yielded more than 250 % increase of the lysine specific productivity without affecting the growth rate or final cell density of the culture [83]. Another important reaction for the production of amino acids derived from the TCA cycle is the PEP-carboxykinase (*pck*) reaction. In contrast to the situation found with pyruvate carboxylase [81], abolition of the PEP carboxykinase activity led to an increase in glutamate production by 440 % and lysine production by 120 % while increasing its activity led to significantly reduced productivities (40 % and 20 %, respectively) [84].

### Streptomyces spp

The fame of streptomycetes as efficient producers of antibiotics started with the discovery of actinomycin in 1940, followed by streptomycin in 1943 [85]. Today, two-thirds of the marketed microbial drugs are produced by streptomycetes. Bacteria of the genus *Streptomyces* have been extensively isolated since 1940 and today the chance of discovering new antibiotics from them is increasing.

Secondary metabolic pathways have been until recently the obvious choice for investigations of strain manipulation and yield improvement in *Streptomyces*. During the last years, genetic manipulation of biosynthetic pathways offered new tools and perspectives. The Embden-Meyerhof-Parnas (EMP) pathway, the PP pathway and the TCA cycle are present in a number of *Streptomyces* species [86] but until recently, the enzymes controlling the primary metabolic pathways have been rather ignored. The productivity of secondary metabolites is mainly determined by the availability of biosynthetic precursors (e.g. acetyl-CoA and malonyl-CoA). Primary metabolism supplies these precursors and therefore, identification and engineering of the enzymes that regulate the carbon flux through the network of reactions of the CCM can increase the availability of precursors [87]. Moreover, metabolic flux rebalancing for precursor-directed biosynthesis provided novel products with improved properties [88].

Optimized metabolic flux distributions were calculated for the production of actinorhodin (ACT) [89] and the calcium dependent antibiotic daptomycin (CDA), both produced by *S. coelicolor*, for various nutrient limitations [90]. Performing metabolic flux analysis, Kim et al. [89] found that CDA production was concomitant with growth and production in batch culture was affected by the oxidative branch of the PP pathway, the shikimate biosynthesis, the oxoglutarate fluxes and nitrogen assimilation. Metabolic flux analysis in chemostat cultures of *S. lividans* grown on glucose or gluconate showed increased carbon flux through glycolysis and the PP pathway while the synthesis of both ACT and undecylprodigiosin (RED) was inverse to the flux through the PP pathway [91]. Deletions of either of two genes *zwf1* and *zwf2* coding for isoenzymes of the initial enzyme of the PP pathway G6PDH, resulted in more efficient utilization of glucose via glycolysis and increased acetyl-CoA precursor pools that led to four-fold increased production of both ACT and RED by *S. lividans*[92].

Ryu and co-workers [93] investigated the roles of key enzymes in central carbon metabolism in the context of increased ACT production by *S. coelicolor*. Genes encoding G6PDH (either *zwf1* or *zwf2*) and phosphoglucomutase (*pgm*) were deleted and those for the acetyl-CoA carboxylase (ACCase) were overexressed. G6PDH encoded by *zwf2* was found to play a more important role than the encoded by *zwf1* in determining the carbon flux to ACT. The *pgm*-deleted mutant produced abutant glycogen but was impaired in ACT production. Finally, overexpression of ACCase resulted in more rapid utilization of glucose and increased efficiency of its conversion to ACT. It was concluded therefore that the carbon storage metabolism plays a significant role in precursor supply for ACT production and that manipulation of the CCM can lead to increased ACT production by *S. coelicolor*.

The first reported application of genetic engineering to channel precursor flux to improve clavulanic acid production by *S. clavuligerus* was that by Li and Townsend [94]. Clavulanic acid biosynthesis is initiated by the condensation of l-arginine and d-glyceraldehyde-3-phosphate (G3P). Two genes (*gap1* and *gap2*) whose protein products are distinct glyceraldehyde-3-phosphate dehydrogenases (GAPDHs) were inactivated in *S. clavuligerus* by targeted gene disruption. A two-fold increase in production of clavulanic acid was obtained when *gap1* was disrupted, and reversed by complementation. Addition of arginine to the cultured mutant further improved clavulanic acid production giving a greater than two-fold increase over wild type, suggesting that arginine became limiting for biosynthesis.

### Lactococcus lactis and other lactic acid bacteria

Apart from their use as starter cultures in the industrial manufacture of fermented food products, lactic acid bacteria (LAB) are also used in a variety of industrial applications, e.g. production of lactic acid, antimicrobial peptides, stereoisomers of lactic acid, high-value metabolites involved in flavor, texture or health applications and probiotic products. Their industrial importance along with some characteristics, such as their small genome size (~2-3 Mb) and simple energy and carbon metabolism, make them promising targets of metabolic engineering strategies. Such strategies have mainly focused either on rerouting of pyruvate metabolism to produce important fermentation end products [95,96] or on the complex biosynthetic pathways leading to the production of exopolysaccharides and vitamins [97,98]. Attempts to manipulate the central carbon metabolism in these bacteria are rather limited compared to the above.

**Figure 2:**
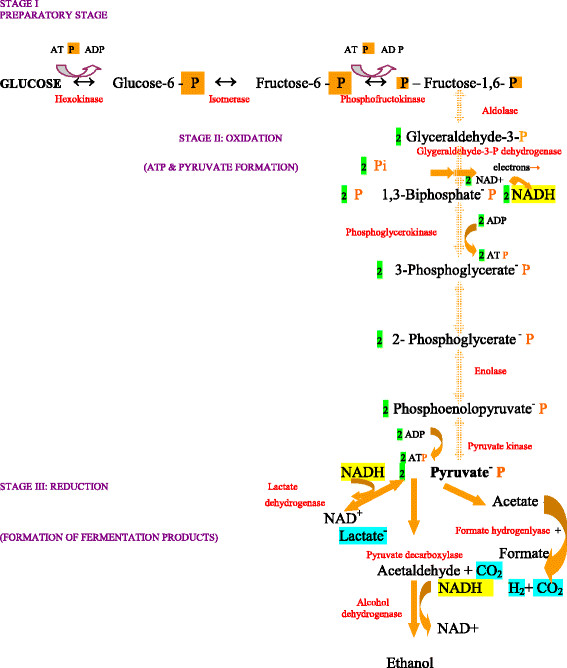
**Glycolysis, the sequence of enzymatic reactions in the conversion of glucose to pyruvate and finally, to fermentation products. **In red letters, are the enzymes involved. Highlighted, are the components exchanged between oxidation or reduction reactions. The number of the produced molecules is given, highlighted in green.

Among LAB, *Lactococcus lactis* is by far the most extensively studied organism. The relative simplicity of *L. lactis* metabolism that converts sugars to pyruvate via the glycolytic pathway (Figure 2) generating energy through substrate level phosphorylation, the availability of numerous genetic tools [99] and the knowledge of its complete genome sequence [100], make it an attractive target for metabolic engineering and the development of effective cell factories [101].

*L. lactis* shows homolactic metabolism when growing in rapidly metabolized sugars with more than 90 % of the metabolized sugar being converted to lactic acid. Deviation from homolactic fermentation is observed under aerobic conditions [102,103] or during the metabolism of galactose or maltose [104,105]. Regulation of glycolysis and the shift between different fermentation modes have attracted considerable attention and have been extensively studied [106-110]. Results reported by Andersen et al. [111] concerning the role of phosphofructokinase (PFK) on glycolytic flux in *L. lactis*, show that this enzyme plays an important role since glycolytic and lactate fluxes were decreased proportionally by a two-fold reduction of PFK activity. A key role was also attributed to PFK with regard to the glycolytic flux control by Neves et al. [103]. Papagianni and co-workers [112] showed that the control of the glycolytic flux resides to a large extend in processes outside the glycolytic pathway itself, like glucose transport and the ATP consuming reactions, and allosteric properties of key enzymes like PFK have a significant influence on the control. Extending their work, Papagianni and Avramidis [112,113] constructed *L. lactis* strains with altered PFK activity, by cloning the *pfkA* gene from *Aspergillus niger* or its truncated version *pfk13* that encodes a shorter PFK1 fragment, and studied the effects of increased PFK activity on the glycolytic capacity of *L. lactis* and lactic acid production. The results demonstrated the direct effect of PFK activity on the glycolytic flux in *L. lactis* since a two-fold increase in specific PFK activity (from 7.1 to 14.5 U/OD_600_) resulted in a proportional increase of the maximum specific rates of glucose uptake (from 0.8 to 1.7 μMs^-1^ g CDW^-1^) and lactate formation (from 15 to 22.8 g lactate (g CDW)^-1^ h^-1^).

It has been shown by Luesink et al. [114] that the glycolytic flux in *L. lactis* is also affected by the carbon catabolite protein (CcpA) which besides its role in catabolite repression also regulates sugar metabolism through activation of the *las* operon (encoding the glycolytic enzymes PFK and PYK, and LDH). Inactivation of the lactococcal *ccpA* gene resulted in a strongly reduced expression level of the *las* operon genes that resulted in a shift from homolactic to mixed acid fermentation [114]. The unique to LAB CcpA-mediated activation of the *las* operon genes has also been observed in *Lactobacillus plantarum* and *Streptococcus thermophilus*[99].

**Figure 3:**
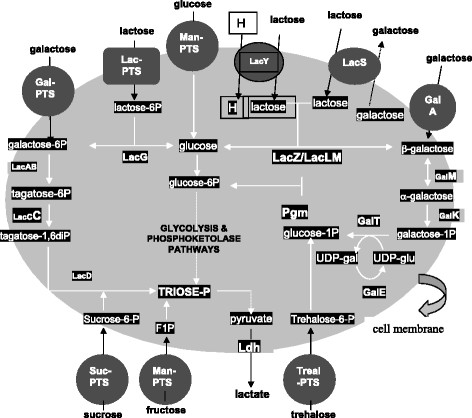
**Metabolic pathways involved in carbohydrate metabolism in** ***Lactococcus lactis.***

Because of its presence in milk and milk-based industrial media, lactose metabolism has been given considerable attention. In many lactococcal strains, the genes involved in lactose metabolism (*lac* operon) are plasmid encoded and the lactose mini-plasmid pMG820 is the best described so far [115]. The plasmid contains the genes involved in the lactose-PTS transport (*lacEF*), lactose-6P hydrolysis (*lacG*) and the tagatose-6P pathway (*lacABCD*) (Figure 3). In 1985, Thompson et al. [116] constructed by classical mutagenesis a double mutant of *L. lactis* that lacked both the glucokinase gene (*glk*) and the mannose-PTS system involved in glucose import. Growth of that strain on lactose involved the conversion of the galactose-6P moiety via the tagatose-6P pathway while the glucose moiety of the disaccharide remained unphosphorylated and secreted. The lack of the mannose-PTS in that strain prevented the subsequent utilization of the secreted glucose. Although the exact mechanism of the engineering is unclear because of the application of random mutagenesis, this early work by Thompson et al. [116] shows how the galactose and glucose metabolism in lactose grown *L. lactis* can be completely separated.

Engineering of carbon distribution between glycolysis and sugar nucleotide biosynthesis is of special importance for the production of UDP-glucose and other activated sugars that serve as precursors for exopolysaccharide (EPS) biosynthesis. EPS from LAB have been given considerable attention recently as they contribute to the development of texture in fermented products; they provide health benefits to consumers (prebiotics) and also protect their producer organisms against bacteriophages and various environmental stresses [99]. UDP-glucose, the most important precursor in EPS biosynthesis, is generated from the glycolytic intermediate glucose-6P by a phosphoglucomutase (encoded by the *pgmU* gene). The intermediate product glucose-1P serves as building block for polymers and it is converted to UDP-glucose by a phosphorylase (encoded by the *galU* gene). Metabolic engineering strategies aiming at overproduction of UDP-glucose have focused on overexpression of the pathway involved genes *galU* and *pgmU*[117,118]. However, decreased activity levels of the key glycolytic enzyme PFK resulted in increased UDP-glucose formation in *L. lactis* grown on glucose [117] but not on lactose [111], the latter indicating that the type of carbohydrate might affect the pathway flux.

## Conclusions

As shown in this review, significant improvement in engineering the central carbon metabolism in industrially important bacteria has been obtained during recent years. The examples presented, exemplify the power of metabolic engineering by applying different methods and approaches, e.g. inactivation, overexpression, redox engineering or even in some cases engineering global control. The quantitative assessment of metabolic fluxes through the central carbon metabolism and of the flux distribution at metabolic branch points, provide detailed information not only for single reactions but also for whole pathways of a variety of bacteria. This information is expanding rapidly by the use of genome-based approaches to new areas and the discovery of novel metabolites especially in the case of *Streptomycetes* and lactic acid bacteria. However, there is still lack of knowledge regarding the prediction of responses of these bacteria to various changes and different conditions. The goal remains to have a global overview of the metabolism of producer strains and accordingly to proceed in the construction of robust strains capable of efficiently producing the desired metabolites. In this view, it is expected that developing System Biology approaches that take into account the gathered information on central carbon metabolism regulation and connect it to global regulation along with improved computational approaches, will provide the opportunities to create efficient cell-factories tailor-made for the production of industrially important metabolites.

## Competing interests

The author declares that she has no competing interests.
